# Metagenomic insights into soil microbial diversity and antibiotic resistance genes in pristine karst tiankeng ecosystems

**DOI:** 10.1128/msphere.00348-25

**Published:** 2025-10-13

**Authors:** Cong Jiang, Yuqing Wu, Changchun Qiu, Sufeng Zhu, Yiyi Zhang, Wei Shui

**Affiliations:** 1 College of Environment and Safety Engineering, Fuzhou University 12423, Fuzhou, China; 2 Ecology and Nature Conservation Institute, Chinese Academy of Forestry 74640, Beijing, China; 3 Geography Department, McGill University 5620, Montreal, Canada; University of Nebraska Medical Center College of Medicine, Omaha, Nebraska, USA

**Keywords:** karst tiankeng, primitive habitat, antibiotic resistance gene, metagenomics, negative terrain

## Abstract

Surveys of microorganisms and antibiotic resistance genes (ARGs) in edaphic systems have centered on those in human-impacted environments, with relatively little information from primitive environments. The karst tiankeng (also known as sinkholes) is the largest negative terrain on the earth’s surface, and the trapped terrain keeps the interior relatively pristine. In this study, three of the most representative tiankeng types (severely, moderately, and non-degraded tiankengs) were selected, and microbial composition, function, and their association with ARGs were determined using metagenetic techniques. The dominant phyla in karst tiankengs were *Proteobacteria*, *Actinobacteria*, and *Acidobacteria*; the dominant archaea were *Crenarchaeota*; and the dominant fungi were *Ascomycota*. The non-degrade tiankeng maintains a complex and stable microbial network. The major functional profiles of the microorganisms are involved in amino acid metabolism and carbohydrate metabolism. A total of 145 ARGs were annotated, and the dominant ARGs in karst tiankeng were *CeoB*, *AcrB*, and *MexF. Paraburkholderia*, *Rhodococcus*, *Bradyrhizobium,* and *Agromyces* were the main hosts of ARGs in karst tiankengs. Compared with ARGs, microorganisms were more influenced by soil factors. These results provide a novel insight into microbes and ARGs in unexplored karst tiankeng ecosystems.

**IMPORTANCE:**

Currently, knowledge regarding the origin of antibiotic resistance genes (ARGs) in pristine soil environments remains limited, with some potentially linked to ancestral genetic diversity. In this study, metagenomics was employed to investigate the distribution of ARGs across nine relatively pristine karst tiankengs. We identified the predominant microbial communities and prevalent types of ARGs within these tiankengs. Soil factors primarily influenced the microbial community structure but had little effect on ARGs. This study offers insights for in-depth research on the microbial composition and risk assessment of antibiotic resistance genes within pristine karst tiankeng ecosystems.

## INTRODUCTION

Antibiotic resistance genes (ARGs) are present in various ecosystems and are a natural defense mechanism used by environmental microorganisms to combat the harmful effects of antibiotics (1). Due to the long-term and massive use of antibiotics, the production and spread of ARGs have accelerated, making ARGs become a new type of environmental pollutant, leading to a global health crisis, and addressing the increasingly severe antimicrobial resistance crisis has become a global challenge (2, 3). At present, most of the research on ARGs focuses on areas with high levels of antibiotic resistance, such as medical wastewater (4), wastewater treatment plants (5), and livestock (6), while ARGs in primitive environments that are either undisturbed or less disturbed by human activities should not be ignored. Many pristine environments have been identified to be repositories of ARGs, such as glaciers (7), grasslands (8), mountain marshes (9), and Antarctica (10). Previous studies have shown that ARGs can be spread macroscopically through physical and biological agents such as wind (11) and migratory birds (12), and microscopically through airborne bacteria (13) and mobile genetic elements (MGEs) (14), without interference from human activities. Identifying the origin and characteristics of ARGs in pristine natural environments can help us understand the background levels and types of ARGs at broader geographic scales. This also aids in tracing the evolution of drug-resistant pathogens, which is critical for assessing the potential risk of human presence in these pristine environments. This also aids in tracing the evolution of drug-resistant pathogens, which is critical for assessing human presence risks.

Karst is one of the world’s leading landforms, covering about 20% of the land area. China is the country with the largest and most widely distributed karst area, covering 3.44 million square kilometers, accounting for about 1/3 of the country’s land area (15). As the largest negative terrain on the surface, the karst tiankeng has the characteristics of huge volume, steep cliff walls, plane width and depth of 100 meters to hundreds of meters, and the bottom is connected to an underground river. Karst tiankengs are often located in remote areas, and the isolation of vertical walls makes the tiankengs maintain an independent original habitat, which is a natural complex of geology, climate, soil, flora and fauna, and microorganisms (16, 17). Our previous research has confirmed that tiankengs are an important “biodiversity conservation bank” and “unique species gene bank” (18). The development stages of karst tiankengs, categorized into severely, moderately, and non-degraded states, are shaped by distinct temporal, spatial, and environmental factors, which, in turn, influence the formation and characteristics of underground forest ecosystems (19). These three stages fully cover the core aspects of the geological structure, hydrological trajectories, and ecological succession of the karst system, providing an irreplaceable natural laboratory for understanding the entire life cycle of karst landform (20). Karst areas are known to be ecologically fragile and vulnerable to human activities, and these areas have experienced severe environmental degradation. Studying ARGs in karst tiankeng ecosystems presents a unique opportunity to explore the natural dynamics of antibiotic resistance in environments minimally influenced by human activities. These ecosystems, characterized by their isolated and pristine nature, serve as ideal settings for understanding the baseline distribution and evolution of ARGs in microbial communities. Metagenomics techniques, which involve the direct sequencing and analysis of microbial DNA in environmental samples, have revolutionized the understanding of microbial diversity and its ecological roles. They have become a powerful tool for understanding the diversity, function, and ecology of microbial communities in a variety of environments (21, 22). Metagenomics expands the utilization space of microbial resources, and ARGs can be traced to the corresponding microbial communities, which provides an effective tool for the study of environmental microbial communities and ARGs (23). In this study, we sampled the tiankeng group in Yunnan Province using metagenomics technology to detect microbial diversity and function, ARGs diversity and hosts, and environmental factors affecting the distribution of microorganisms and ARGs. We have selected the three most typical types of karst tiankengs, representing the different stages of development of karst tiankengs (20). The results of this study will help to improve our understanding of the background level and types of ARGs in native soils and provide a theoretical reference for predicting microbial composition and the emergence of antibiotic resistance in karst negative terrain areas.

## MATERIALS AND METHODS

### Study area and soil sample collection

Zhanyi Tiankeng Group (25°35′–25°57′ N, 103°29′–103°39′ E; Fig. S1) is located in the eastern part of Yunnan Province. The rock is mainly composed of carbonate rocks. The soil is mainly red soil, which is iron-rich aluminum soil formed in tropical-subtropical climates. It typically exhibits acidity, pronounced weathering features, and a complex mineral composition (24). The region has a subtropical plateau monsoon climate, with an average annual temperature of 13.8–14.0°C, annual rainfall of 1,073.5–1,089.7 mm, annual evaporation of 2,069.1 mm, and relative humidity of 71%. Zhanyi Tiankeng group is one of the most representative Tiankeng groups in China, with dozens of tiankengs of different sizes and degrees of degradation, and a complete evolutionary chain (25). Three typical tiankengs were selected: non-degraded tiankengs (NDT), moderately degraded tiankengs (MDT), and severely degraded tiankengs (SDT). The classification criteria for the three typical tiankengs were described by Chen et al. (26). NDT features steep vertical cliffs and immense spatial dimensions, with their bases directly connected to active subterranean river systems. These formations create enclosed microclimates that preserve primeval forest communities and relict species. MDT refers to formations where wall integrity is compromised, manifesting as localized collapses and reduced or seasonally interrupted underground water flows though core geological characteristics (e.g., main cliff structures) remain partially intact. SDT exhibits extensively fractured geological structures with obscured contours, accompanied by complete diversion or desiccation of underground rivers. SDT exhibits greater vulnerability to anthropogenic activities—particularly grazing and farming—than NDT or MDT.

**Fig 1:**
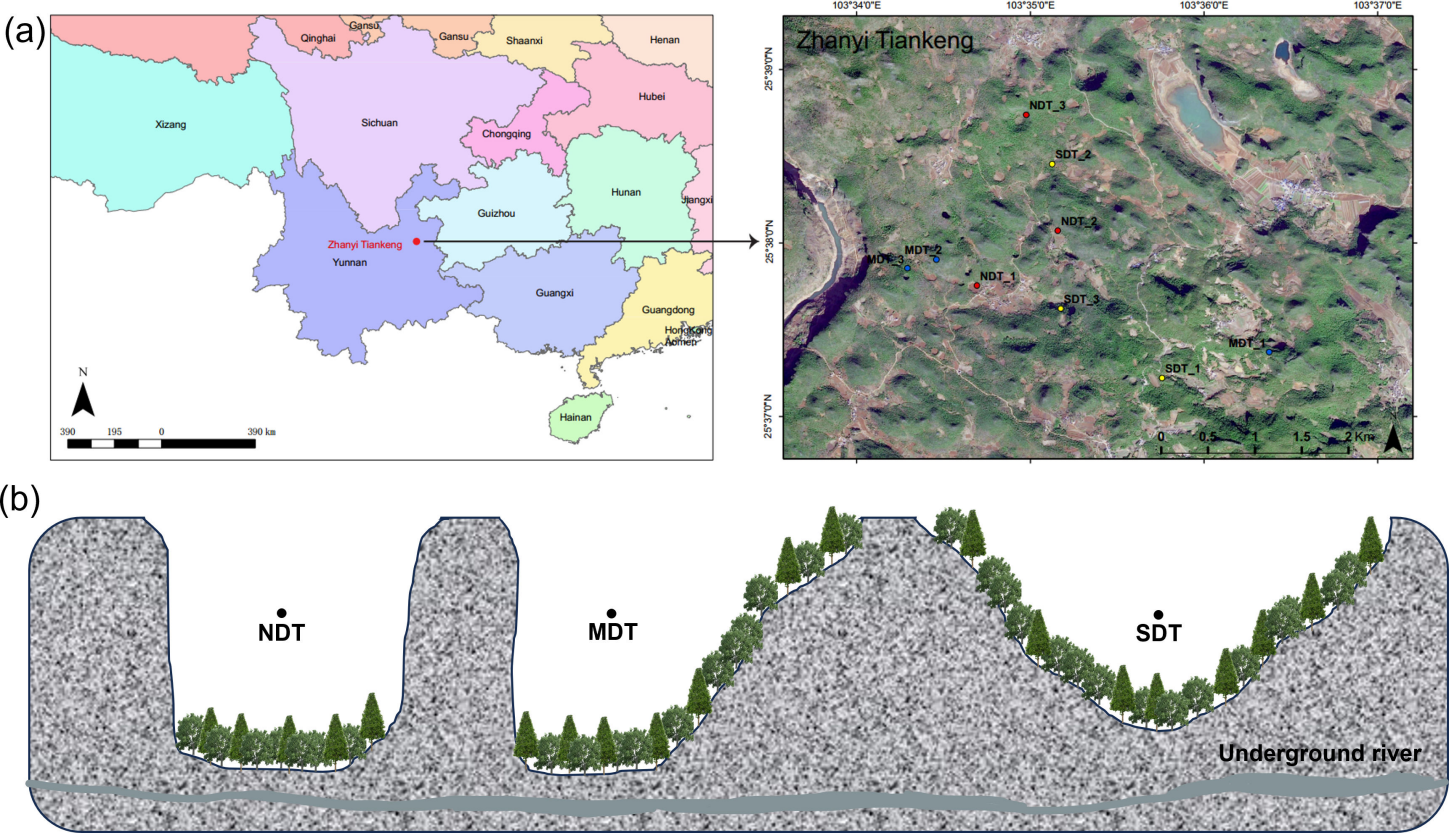
Distribution of soil sampling points in Zhanyi karst tiankeng (**a**) and schematic cross-sectional diagram of karst tiankeng (**b**). NDT, non-degraded tiankengs; MDT, moderate degraded tiankengs; SDT, severely degraded tiankengs.

The soil samples were collected from three types of karst tiankengs (severely, moderately, and non-degraded tiankengs), each type comprising three individual tiankengs (Fig. 1). In each single tiankeng, we established three 10 × 10 m^2^ plots. For each plot, soil cores from 0 to 20 cm were collected using the five-point sampling method and mixed to a composite sample, resulting in a total of 27 soil samples. After removing the roots and stones, each homogenized soil sample was divided into two subsets, one subset was used for soil physicochemical properties analysis, and the other subset was stored at −80°C for the metagenomic analysis.

### Metagenomic sequencing

In total, 0.5 g soil was used to extract the DNA by CTAB methods. DNA purity and concentration were tested by Agilent 5400. The NEBNext UltraTM DNA Library Prep Kit for Illumina (NEB, USA, Catalog#: E7370L) was used to construct the sequencing library. The qualified libraries were pooled equally and sequenced on Illumina platforms with PE150 strategy according to standard protocols by Wekemo Tech Group Co., Ltd. Shenzhen, China. The raw data of archaea, bacteria, fungi, and viruses in soil samples were obtained by metagenomic sequencing. The raw sequencing data were preprocessed using Kneaddata software (Version 0.7.4) to obtain reliable data (27). By using Bowtie2 software (Version 2.3.5.1) (parameter: very sensitive) to get clean data. For each soil sample, the default parameters of MEGAHIT (Version 1.2.9) were selected for assembly, and the contigs of the samples were obtained. All contigs were summarized, and fragments below 500 bp were filtered out for statistical analysis and subsequent gene prediction (28). Species contained were identified by the Kraken2 (Version 2.0.7-beta) and the self-build microbial database, with actual species relative abundance predicted using Bracken (Version 2.0) (29). The eggnog-mapper software (Version 2.1.7; based on DIAMOND) was used to compare the non-redundant protein sequences to the KEGG database (https://www.kegg.jp/), and the functional annotation information of the proteins was obtained. The DIAMOND software (Version 0.8.22) was used to align the redundant genes to the CARD database (https://card.mcmaster.ca/), and the annotation information of resistance genes was obtained (30). According to the abundance and annotation information of the non-redundant genes, the abundance of the non-redundant genes annotated to the same gene was summed, and the redundant genes that failed to align were screened out to obtain the relative abundance of ARGs.

### Soil physicochemical properties

Soil water content (SWC) was determined by drying fresh soil to constant weight at 105°C. Soil organic carbon was determined by the wet oxidation method, and soil organic carbon content was multiplied by the coefficient 1.724 to obtain soil organic matter (SOM). Soil dissolved organic carbon (DOC) was determined by mixing 3 g of soil with deionized water at a ratio of 5:1, and the extract was detected by TOC analyzer. Soil total nitrogen (TN) was determined by the Kjeldahl method. Soil available nitrogen (AN) was determined by the alkaline diffusion method. Soil total phosphorus (TP) was determined by the alkali fusion-Mo-Sb anti-spectrophotometric method. Soil available phosphorus (AP) was determined by the sodium hydrogen carbonate solution-Mo-Sb anti-spectrophotometric method. Soil calcium and magnesium contents were determined by inductively coupled plasma atomic emission spectrometry (ICP-AES). The soil pH was determined by extracting the soil with ultrapure water and drying the soil (water-soil ratio 2.5:1).

### Statistical analysis

The non-metric multidimensional scaling (NMDS) based on average Bray-Curtis dissimilarity values was used to visualize the beta diversity, and the significance of difference was calculated by similarity analysis (ANOSIM). The relationship between microbial species and ARGs was assessed using Spearman correlation analysis and visualized via Cytoscape (Version 3.10.3) (31). The natural connectivity was used to detect the robustness of microbial networks, and analytical procedures followed previous studies (32). The correlation between soil physicochemical properties and microbial and ARGs was evaluated using the Pearson correlation coefficient (9). Statistical analyses and draws were conducted by R (Version 4.3.2).

## RESULTS

### The soil physicochemical properties of karst tiankeng

The soil physicochemical properties differed among the three types of karst tiankeng (Table 1). The contents of SOM and TN were significantly higher in MDT (*P* < 0.05), and MDT and NDT showed no significant differences. The contents of TP were markedly lower in SDT. The soil of the karst tiankeng was acidic, and significant differences were observed among NDT, MDT, and SDT. The DOC, AN, AP, Ca, Mg, and SWC were not significantly different among the three types of karst tiankeng.

**TABLE 1:** Summaries of soil physicochemical properties of karst tiankeng^*a*^

Karst tiankeng	SOM (g/kg)	DOC (mg/kg)	TN (g/kg)	AN (mg/kg)	TP (mg/kg)	AP (mg/kg)	Ca (g/kg)	Mg (g/kg)	SWC	pH
SDT	63.69 ± 29.22b	345.65 ± 93.50a	3.10 ± 0.76b	256.26 ± 89.99a	539.44 ± 96.89b	9.34 ± 5.78a	1.65 ± 1.30a	0.10 ± 0.06a	0.28 ± 0.06a	6.28 ± 0.42b
MDT	89.93 ± 27.44a	332.38 ± 58.77a	4.22 ± 1.30a	275.22 ± 116.73a	659.26 ± 116.81a	11.49 ± 6.35a	1.61 ± 0.88a	0.18 ± 0.11a	0.31 ± 0.04a	6.81 ± 0.31a
NDT	76.92 ± 19.54ab	315.09 ± 73.07a	3.56 ± 0.99ab	292.14 ± 33.31a	648.58 ± 106.71a	13.03 ± 7.25a	1.53 ± 0.91a	0.17 ± 0.09a	0.27 ± 0.05a	6.82 ± 0.39a

^*a*^ SOM, soil organic matter; DOC, dissolved organic carbon; TN, total nitrogen; AN, available nitrogen; TP, total phosphorus; AP, available phosphorus; SWC, soil water content; NDT, non-degraded tiankengs; MDT, moderate degraded tiankengs; SDT, severely degraded tiankengs; Values are mean ± standard error. Different lowercase letters indicate significant relationships at 0.05 levels.

### The soil microbial community composition and structure of karst tiankeng

There are 19,038,786–23,153,381 clean reads for each soil sample. Contigs evaluation found that the average number of contigs longer than 1,000 bp was 2,303,306, and the N50 was 723 bp. The coverage values of all samples exceeded 97%, indicating that the metagenomic sequencing was adequate to almost cover all microorganisms. It could thoroughly reflect the microbial community composition of karst tiankeng.

**Fig 2:**
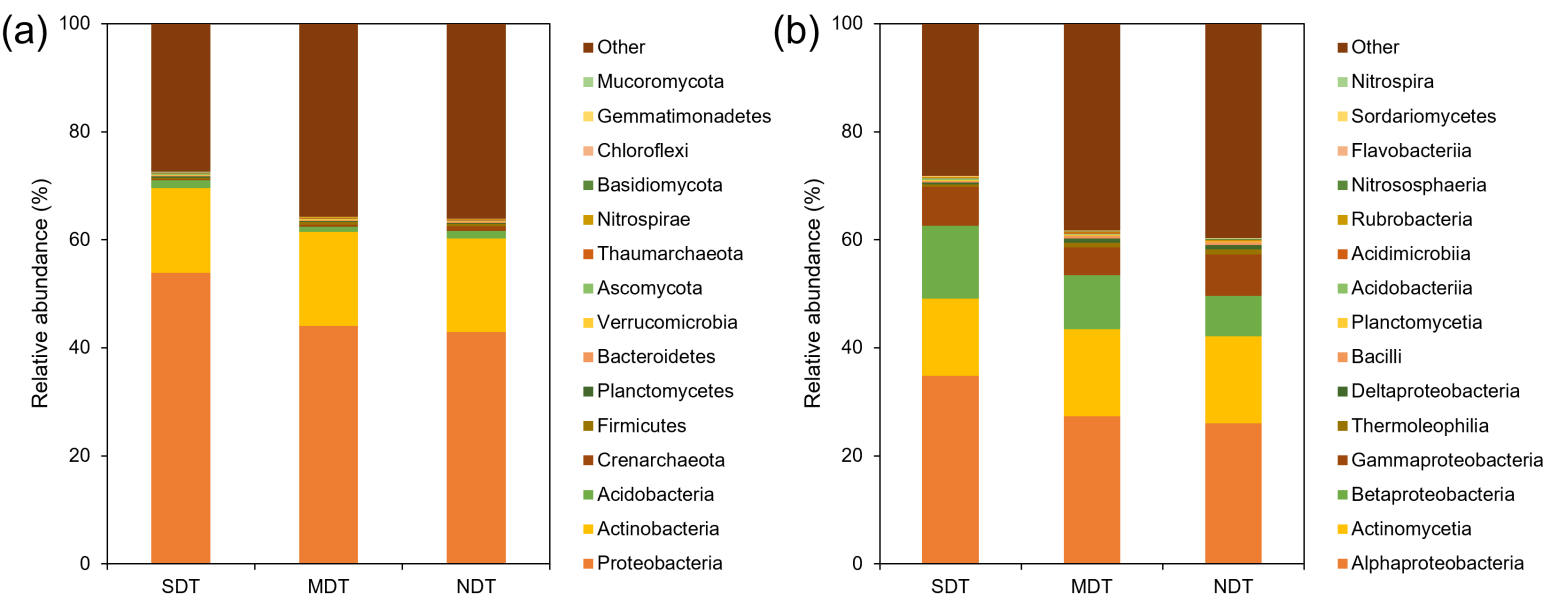
The composition of the microbial community of karst tiankeng soils (**a**) at phylum level and (**b**) at class level.

Microorganisms annotated in the karst tiankeng soil included 4 domains, 10 kingdoms, 61 phyla, 106 classes, 245 orders, 502 families, 1,235 genera, and 4,740 species. Among them, bacteria (98.67%–99.18%) were dominant, followed by archaea (0.27%–1.13%), and fungi (0.19%–0.49%). There was no significant difference in the dominant microbial composition among these three types of karst tiankeng. At the phylum level (Fig. 2a), the dominant bacteria included *Proteobacteria* (42.92%–53.88%), *Actinobacteria* (15.70%–17.41%), and *Acidobacteria* (0.98%–1.44%); the dominant archaea: *Crenarchaeota* (0.19%–0.87%). At the class level (Fig. 2b), the dominant bacteria included: *Alphaproteobacteria* (26.03%–34.85%), *Actinomycetia* (14.22%–16.12%), and *Betaproteobacteria* (7.50%–13.51%); the dominant archaea: *Nitrososphaeria* (0.03%–0.15%); and the dominant fungi: *Sordariomycetes* (0.04%–0.11%). At the species level, the Venn diagram results exhibited that 4,740 species of soil microorganisms shared in the three types of karst tiankeng, of which 702 microbes were unique to the SDT, with the highest diversity, and only 448 microbes were unique to MDT, with the lowest diversity (Fig. S1).

The analysis of similarities (ANOSIM) (*r* = 0.352, *P* = 0.001) showed that the soil samples significantly differed among the three types of karst tiankeng (Fig. S2). The NMDS showed that the soil microbial communities in SDT and MDT clustered closely apart from those in NDT (Fig. S3).

**Fig 3:**
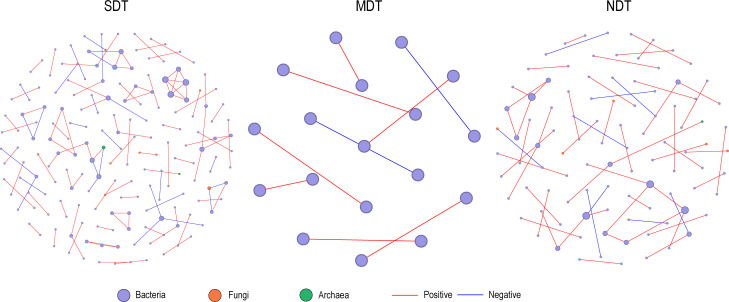
Co-occurrence patterns of the microbial community of karst tiankeng soils.

The co-occurrence network analysis showed that tiankeng degradation significantly changed the soil microbial interactions. Specifically, SDT and NDT maintained a more complex network structure than MDT (Table S1). More positive connections were detected in all three networks, suggesting that soil microbes in tiankengs rely on promotion effects to maintain their survival (Fig. 3). Furthermore, the natural connectivity analysis was used to test the robustness of the soil microbial networks in karst tiankengs under different degradation levels. The results demonstrated a significant impact of karst tiankengs degradation on the robustness of the microbial network. Despite the MDT having higher robustness values than the NDT and SDT, the natural connectivity value of MDT fluctuated more sharply with the increase in the proportion of removed nodes (Fig. S4).

### The soil microbial community functional of karst tiankeng

Microbial functions were also annotated based on the KEGG database (Fig. S5). Results showed that Metabolism (75.01%–75.24%) was the most abundant microbial function, followed by Genetic Information Processing (7.95%–8.26%) and Cellular Processes (6.07%–6.25%), Human Diseases (5.14%–5.18%), Organic Systems (3.23%–3.28%), and Environmental Information Processing (2.20%–2.26%). Further analysis found that there were 31 pathways involved in the carbon cycle, 11 pathways to the nitrogen cycle, and 14 pathways to the sulfur cycle (Fig. S6). The most carbon and nitrogen cycles were significantly abundant in SDT. Therefore, we concluded that the degree of tiankeng degradation could significantly affect the carbon and nitrogen cycles. This pattern may be closely related to anthropogenic activity, which shows clear signs (e.g., growing crops or grazing) at the bottom of SDT. Methane metabolism was found in three types of karst tiankeng (Fig. S6a), which may be related to the formation of wetlands by seasonal water accumulation at the bottom of karst tiankeng.

### Types of ARGs in karst tiankeng

**Fig 4:**
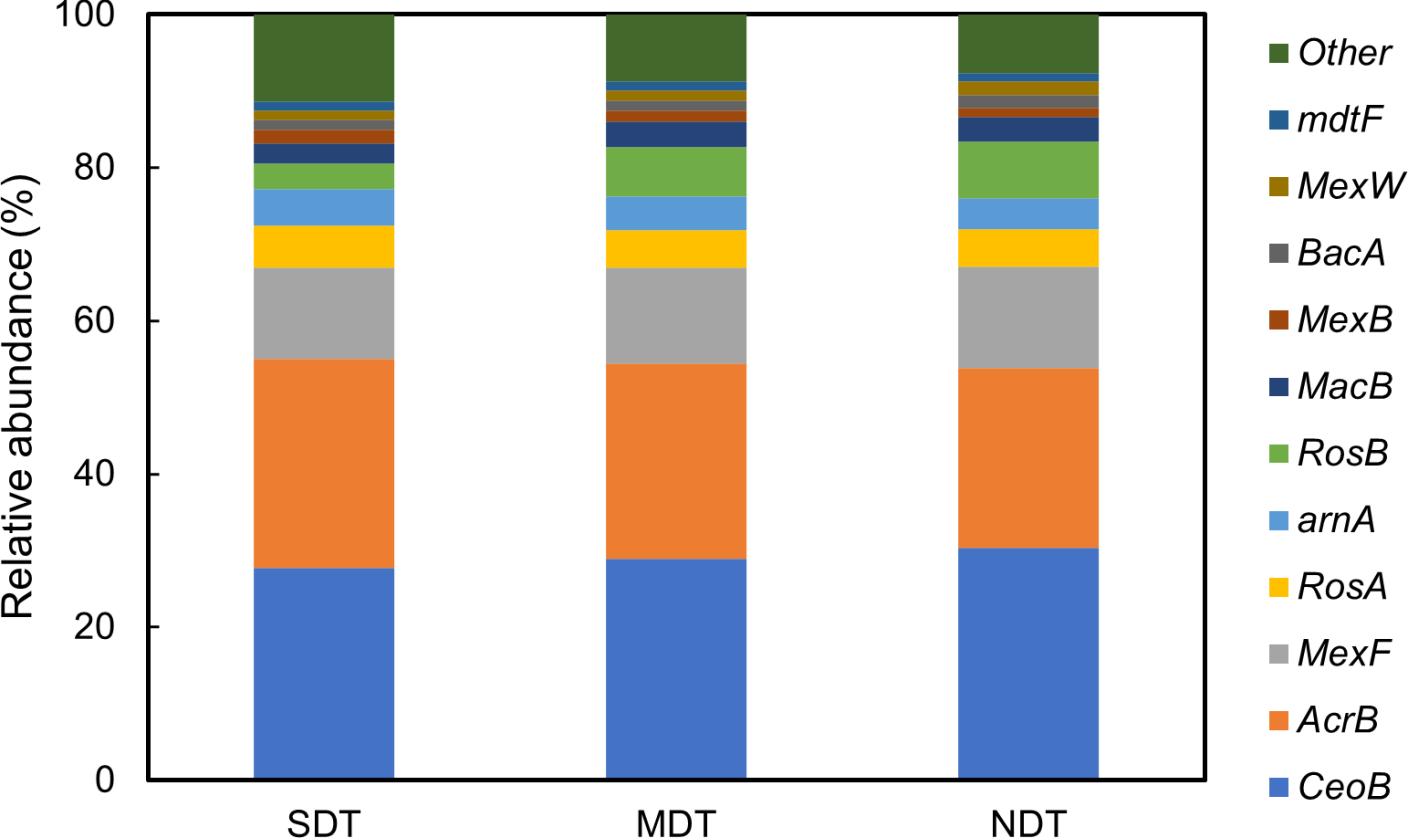
The composition of the ARGs of karst tiankeng soils.

There were 145 ARGs annotated from the soils in karst tiankengs. Among them, chloramphenicol resistance gene (*CeoB*, 27.76%–30.41%) accounted for the highest proportion, followed by macrolide resistance gene (*AcrB*, 23.38%–27.26%), fluoroquinolone resistance gene (*MexF*, 11.89%–13.34%), and fosmidomycin resistance gene (*RosA*, 4.88%–5.54%). Twelve resistance genes were detected exclusively in SDT, including streptomycin resistance gene (*Aph6Ia* and *Aph6Id*), cephalosporin resistance gene (*BL1_acc*, *BL1_ceps*, *BL2be_per*, and *BL3_cphA*), fosfomycin resistance gene (*FosA*), multidrug resistance efflux pump resistance gene (*MexH*), glycylcycline resistance gene (*MexX*), chloramphenicol (*cml_e8*), tobramycin resistance gene (*Aac2I*), and vancomycin (*VanT*) (Fig. 4).

Among these genes detected, the top six most abundant ARG subtypes (e.g., *CeoB*, *AcrB*, *MexF*, *RosA*, *arnA*, *RosB*) accounted for over 80% of the total ARGs in karst tiankeng soils, which were mainly associated with chloramphenicol, multidrug, polypeptides, and trimethoprim. ARGs diversity of the karst tiankeng at level 1 was analyzed using Venn diagram (Fig. S7). The results showed that SDT contained the highest number of unique ARGs (12 subtypes), while MDT had the lowest (3 subtypes).

**Fig 5:**
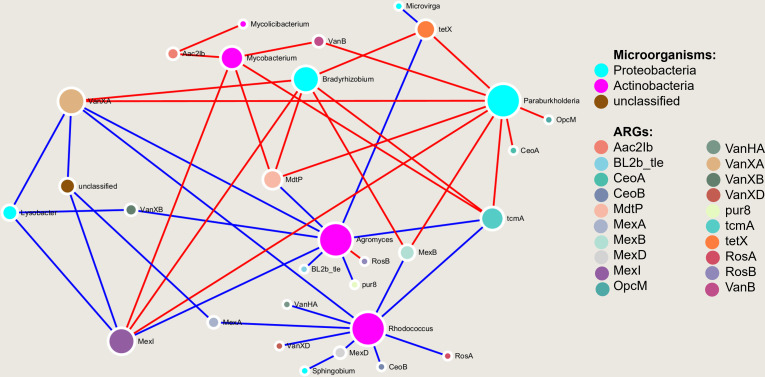
The network among ARGs and microorganisms (red, positive correlation; green, negative correlation).

At a genus level (microorganism) and type level (ARGs), the correlation between the top 30 total abundance of microbial species and ARGs was analyzed based on the Spearman coefficient (Fig. 5). Results showed that *Aac2Ib*, *VanB*, *OpcM*, *CeoA,* and *RosB* were positively correlated with microorganisms. In contrast, the *VanXB*, *MexA*, *VanXD*, *VanHA*, *BL2b_tle, pur8*, *MexD, CeoB,* and *RosA* were negatively correlated with microorganisms. Among them, *VanHA, MexA, VanXD, MexD, CeoB*, and *RosA* were negatively correlated with *Rhodococcus*, which belongs to the *Actinobacteria* bacterial genera. *Paraburkholderia* and *Bradyrhizobium* were subordinated to *Proteobacteria* and were positively correlated with *tcmA, Mexl, MdtP*, and *VanXA*, indicating that they were the key genera that could promote the spread of ARGs in karst tiankeng.

### Correlation between the microorganisms, ARGs, and soil physicochemical properties

**Fig 6:**
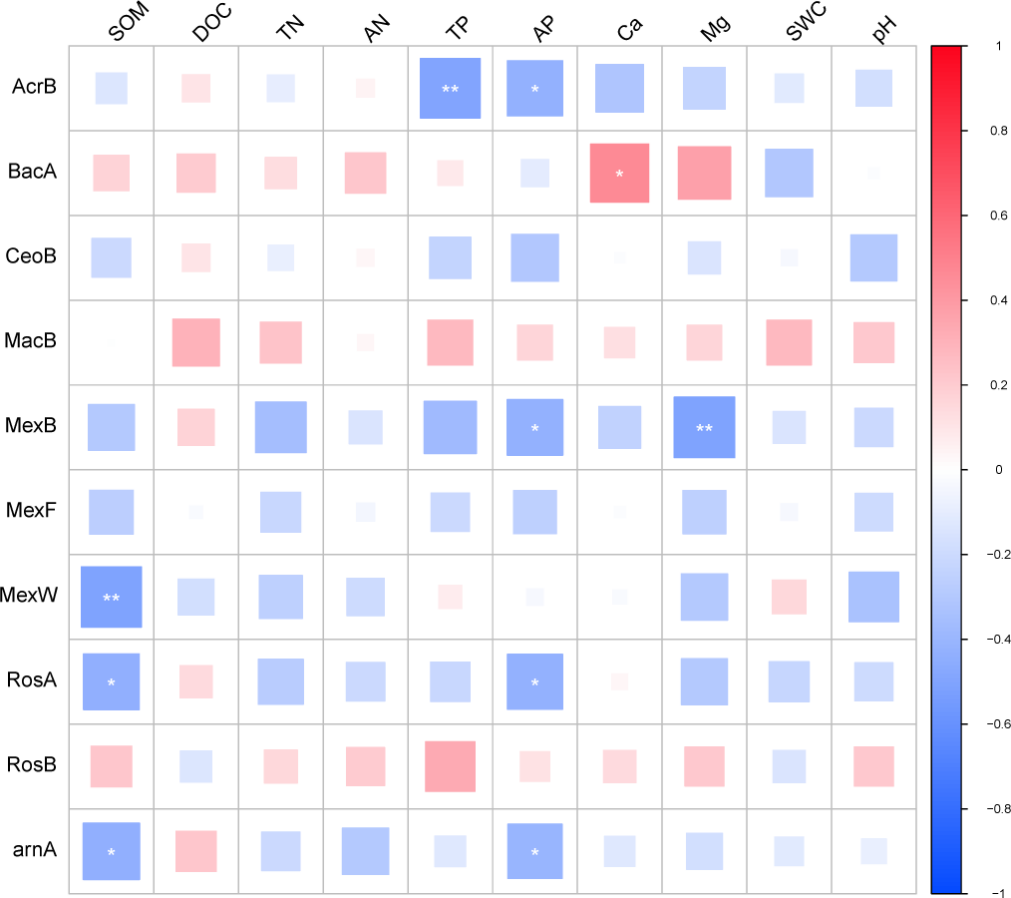
Correlation between ARGs and soil physicochemical properties in karst tiankeng.

In terms of microorganisms (Fig. S8), DOC had a positive correlation with archaea, bacteria, and viruses. SOM had a significant positive correlation with most of other microorganisms, while it had a significant negative correlation with *Steptomycetales* and *Micromonosporales*. TN, AN, and Ca affected bacteria distributions, while AP affected archaea distribution. TP had a significant negative with bacteria and fungi, and significant positive with *Halobacteriales* and *Thermococcales* of archaea. Mg had a significant positive with *Methanobacteriales* and *Micromonosporales*, and negative with *Eurotiales* and *Chaetothyriales*. SWC had little effect on microorganisms. pH had a significant positive with *Nitrososphaerales* and *Sphingomonadales*, and negative with *Chaetohyriales* and *Hyphomicrobiales*. Overall, soil factors had a greater impact on the archaea and bacteria. For ARGs (Fig. 6), SOM was negative with *RosA* and *arnA*, DOC was positive with *MacB*. TN, AN, SWC, and pH did not affect ARGs. TP was negative with *AcrB*, and positive with *RosB*. AP was negative with *RosA*. Ca was positive with *BacA*. Mg was negative with *MexB*.

## DISCUSSION

### Microbial community structure and function in karst tiankengs

The metagenomics studies have helped uncover karst tiankeng microbial and ARGs diversity, providing valuable insights into the genetic makeup of microbes and distribution of soil antibiotic resistance genes in pristine habitats (33). In this study, taxonomically and functionally diverse microorganisms were found in karst tiankengs with a dominance of *Proteobacteria, Actinobacteria, and Acidobacteria*, which is consistent with the previous study of karst land soil (34). *Proteobacteria* play key roles in ecological and phylogenetic values and participate in the oxidation of organic and inorganic substrates (35). The abundant soil nutrients and excellent hydrothermal conditions promote *Proteobacteria* survival well in karst tiankeng habitats. *Actinobacteria* exhibit a strong metabolic ability in low-temperature environment. Karst tiankengs are deep in the surface and have the characteristics of a low temperature and high humidity microclimate and exhibit a trend of decreasing temperature with increasing depth (36). *Acidobacteria* are considered to be involved in nutrient cycles and organic matter decomposition (37). This result suggested that microbes capable of nutrient degradation and possessing higher metabolic activity might survive well in karst tiankengs. However, the same soil microbial taxa with different relative abundances were found in three types of karst tiankeng.

In comparison to bacteria and fungi, there was little research about the archaeal communities in karst ecosystems. *Crenarchaeota* and *Thaumarchaeota* were the most abundant archaea in karst tiankeng. The evidence exhibited that the *Crenarchaeota* were the most abundant archaea in karst caves (38, 39). Karst tiankengs and caves are formed for similar reasons and often coexist, which may explain the similarity in the composition of certain microbial taxa. In addition, *Thaumarchaeota* are considered to be the most common and ubiquitous archaea in wetlands (40). This may be due to the presence of wetlands formed by seasonal water accumulation at the bottom of the karst tiankengs. As key ammonia oxidizers, *Thaumarchaeota* contribute over 40% to ammonia oxidation (24), suggesting archaea play a vital role in the karst tiankeng nitrogen cycle. The most abundant fungi were *Ascomycota*, *Basidiomycota,* and *Mucoromycota. Ascomycota* are considered to be the abundant and diversified eukaryotes in the red soil and involved in the decomposition of organic compounds (41). *Basidiomycota* includes some plant parasite fungi that play a key role in wood degradation and litter decomposition. *Basidiomycota* eukaryote species can store mineral nutrients and water and have a symbiotic relationship with the plant roots (42, 43). *Mucoromycota* are considered to be the most common species in a variety of habitats. As the sub-phylum of *Mucoromycota*, arbuscular mycorrhizal fungi (AMF) are considered to significantly improve plant nutrient uptake (44, 45). These findings suggest that *Ascomycota*, *Basidiomycota,* and *Mucoromycota* were well adapted to the underground forests in karst tiankeng (46).

The complexity and stability of microbial networks vary among karst tiankengs with different degrees of degradation. The previous studies have demonstrated that in severely degraded ecosystems, more complex species interrelationships may be formed to resist environmental disturbances (47, 48). This phenomenon was observed in this study and explained the increased microbial network complexity in SDT compared with MDT (Fig. 3). Due to environmental isolation, the NDT maintains a stable and complex microbial network structure. Our study highlights that karst tiankengs (specifically NDT) provide a unique niche for the survival of soil microorganisms.

In this study, microorganisms were involved in diverse pathways, including the carbon cycle, nitrogen cycle, and sulfur cycle (Fig. S6). *Alphaproteobacteria*, *Bacilli*, *Nitrososphaeria*, and *Nitrospira* were found as the abundant classes in the karst tiankengs and were closely associated with the nitrogen cycle (49–51). The detection of *Deltaproteobacteria* in karst tiankengs indicates their extensive involvement in the sulfur cycle, including abundant sulfate-reducing bacteria, which could utilize various growth substrates (52). The role of water in methane metabolism is pivotal, as it serves as a primary reactant and medium for methanogenic processes. In our study, we found that methane metabolism may be influenced by seasonal wetlands in karst tiankeng ecosystems (Fig. S6a). This finding aligns with previous studies that have demonstrated the critical role of water in shaping methanogenic communities and their metabolic potential (53, 54).

### ARGs types and their correlation with microorganisms

Karst ecosystems have complex geological characteristics, which make them susceptible to pollution by human activities and the rapid spread of pollutants over a large scale. Simultaneously, pristine forest soils exhibit rich ARG diversity, serving as a potential source of resistance traits. Thus, karst tiankengs are an important reservoir for the ARGs (53, 55, 56). As a typical type in the karst ecosystem, karst tiankengs are less disturbed by human activities due to the isolation of vertical cliffs. Consequently, ARGs in such environments are more likely to be close to historical genes, especially in NDT. In contrast, severely degraded tiankengs are more susceptible to external human disturbances, which may change the composition and distribution of ARGs. ARGs in karst tiankengs were dominated by chloramphenicol resistance gene (*CeoB*), macrolide resistance gene (*AcrB*), and fluoroquinolone resistance gene (*MexF*). *CeoB* gene was always frequently observed in relatively pristine environments and popular in the plasmids of various pathogens (57, 58). *AcrB* was a multidrug resistance gene (MRGs) and prevalent and abundant in Tibetan and Antarctic circumstances (58, 59). *MexF* is thought to be widespread in agroecosystems (60, 61). The high abundance of *MexF* was observed in karst tiankengs, which may be due to the complex and highly connected nature of karst water systems (55).

Previous studies have confirmed that microorganisms are innately resistant to antibiotics in nature (62). Antibiotics in nature compete with microorganisms for survival and domesticate surrounding microorganisms to resist environmental stresses (63, 64). In our study, some bacterial taxa belonging to *Proteobacteria* and *Actinobacteria* were the predominant potential hosts for ARGs. *Actinobacteria*-derived ARGs constitute a pivotal component of the environmental resistome, primarily because *Actinobacteria* serve as major producers of antibiotics (65). These microbial taxa are known for their widespread distribution and genetic exchange capabilities and have been confirmed in previous studies (23, 66). One type of ARGs can be carried by a variety of microorganisms. For example, *Mexl* was carried by multiple types of microorganisms. *Agromyces*, *Rhodococcus*, and *Paraburkholderia* coexisted with *tcmA* and *vanXA*, indicating that they may act as potential hosts for these ARGs (Fig. 5). Differences in microbial communities can affect the abundance of potential host microorganisms for ARGs, which will directly affect the abundance and distribution of ARGs (67). Our study observed that there were differences in the composition of microbial communities among the three types of karst tiankengs, especially the differences in abundance of abundant taxa *Proteobacteria* and *Actinobacteria* may be the key factors affecting the distribution pattern of ARGs. The above results further suggested that microorganisms carrying multiple ARGs were susceptible to antibiotic resistance, and the potential environmental risks of ARGs should be closely monitored.

### Influence of environmental factors on the distribution of microorganisms and ARGs in karst tiankengs

Our results showed that the soil physicochemical properties were closely related to the composition of archaea and bacteria. DOC and TP were the major drivers of archaeal community composition. *Methanomicrobiales* and *Methanosarcinales* are types of methanogens that exist in anoxic environments. They are stably distributed across three types of karst tiankengs, which is closely related to the seasonal wetlands within karst tiankengs. DOC is an active carbon component and can be easily utilized by microorganisms (68). In this study, a significant positive correlation between DOC and methanogens was observed, and high concentrations of DOC provided favorable environmental conditions for methanogens to influence the CH_4_ metabolic pathway. TP was the main factor affecting the archaeal community composition (69), corresponding to the results in this study. The unique karst landform of the region enhances groundwater recharge (70), thereby promoting the release of mineral-bound phosphorus and increasing TP (71). Bacterial communities in karst tiankengs were mainly correlated with SOM and TN. Soil nutrients play key roles in driving the soil bacterial community composition (72). Our results showed that soil physicochemical properties were not the key factors affecting the fungal community composition. Previous studies showed that soil characteristics correlate weakly with fungal communities, while plant diversity or types are the major drivers of fungal community diversity (73, 74).

Soil physicochemical properties were identified as critical factors in driving the dissemination of ARGs. SOM is one of the non-negligible soil properties involved in soil biological processes (75). In this study, SOM was negatively correlated with the ARGs. These results supported the previous discovery that the total organic carbon content was negatively correlated with the richness of ARGs (76). Our results emphasize that SOM acted as a key factor in driving the composition of ARGs. TP also served as a key factor related to ARGs (Fig. 6). Previous studies indicated that TP can affect soil microbial community structure (67). pH could influence the transfer of ARGs, such as acidification would reduce the transfer of sulfa resistance genes (77), while alkaline conditions may affect the production of tetracycline resistance genes (78). These findings differ from ours, which may be due to the little variation in pH among the three types of karst tiankeng.

### Conclusion

This study revealed the soil microbial composition, potential functional profiles, and their relationship with ARGs in karst tiankengs and improved our understanding of the ARGs profiles of magnificent underground ecosystems. The dominant phyla in the three types of karst tiankengs were similar and mainly included *Proteobacteria*, *Actinobacteria*, and *Acidobacteria*. The microbial community structures of the non-degraded tiankengs were significantly different from those of other types of tiankengs. The most carbon and nitrogen cycles were significantly abundant in severely degraded karst tiankengs. Chloramphenicol resistance, macrolide resistance, and fluoroquinolone resistance genes were the most abundant antibiotic resistance genes in the karst tiankeng ecosystem. *Paraburkholderia*, *Bradyrhizobium*, *Rhodococcus*, and *Agromyces* were the crucial host microorganisms that promoted the spread of ARGs in karst tiankengs. The soil physicochemical properties mainly affected the microbial community structure, especially archaea and bacteria, rather than ARGs. It is important to note that metagenomics may not fully capture the entire soil microbiota; thus, complementary approaches like targeted amplicon sequencing or metatranscriptomics are warranted. Furthermore, while this study focused on soil physicochemical properties, future research should pay more attention to anthropogenic factors such as land-use practices. To our knowledge, this study is the first to explore the microbial diversity, composition, and potential hosts of ARGs in the karst tiankeng ecosystem, expanding our understanding of the occurrence state of ARGs in relatively primitive ecosystems.

## Data Availability

The DNA sequences were uploaded to the NCBI-SRA under the accession number PRJNA1133678.

## SUPPLEMENTAL MATERIAL

Supplemental material msphere.00348-25-s0001.docx Fig. S1 to S8; Table S1.

ASM does not own the copyrights to Supplemental Material that may be linked to, or accessed through, an article. The authors have granted ASM a non-exclusive, world-wide license to publish the Supplemental Material files. Please contact the corresponding author directly for reuse.
