# Supplementary material for: Metagenomic insights into soil microbial diversity and antibiotic resistance genes in pristine karst tiankeng ecosystems
Source: mSphere. 2025 Oct 13;10(11):e00348-25. doi: 10.1128/msphere.00348-25 (PMC12645922; doi:10.1128/msphere.00348-25)
Supplement: Supplemental material — Fig. S1 to S8; Table S1. [file msphere.00348-25-s0001.docx]

**Supplement Files**


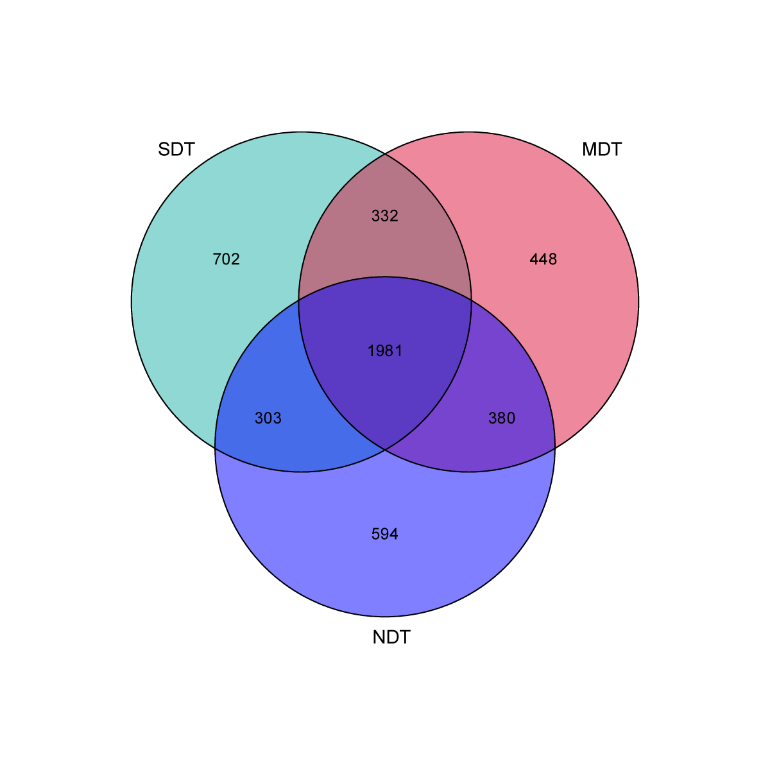


Fig. S1. Venn diagram of microbial community composition of karst tiankeng soils.


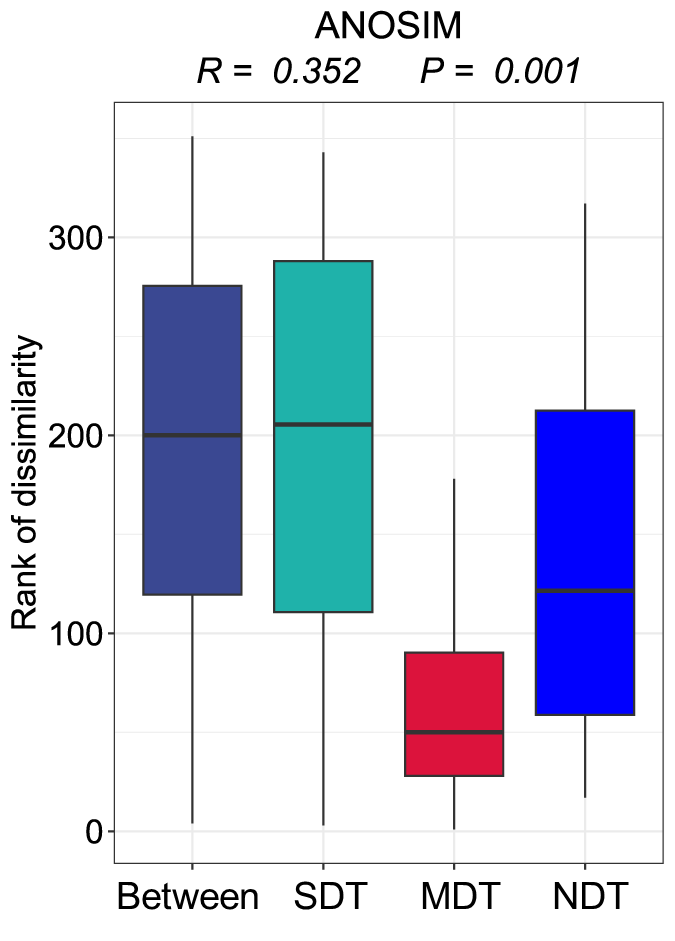


Fig. S2. The analysis of similarities (ANOSIM) of microbial community of karst tiankeng soils.


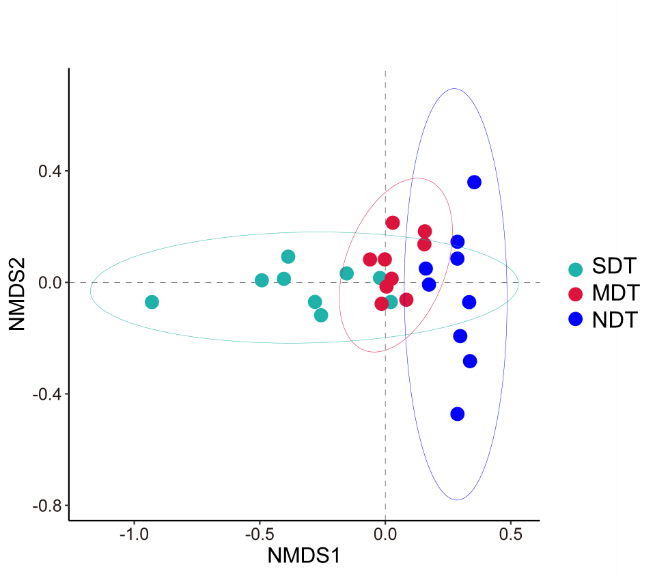


Fig. S3. The non-metric multidimensional scaling (NMDS) of microbial community composition of karst tiankeng soils.


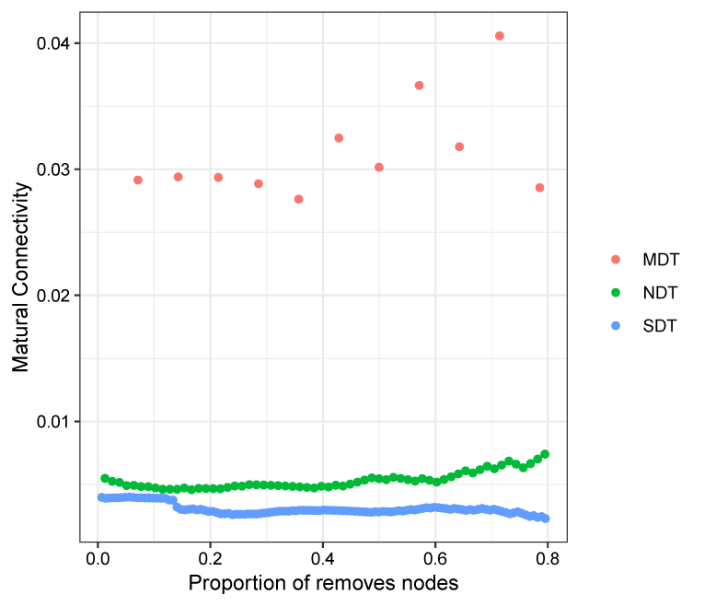


Fig. S4. The natural connectivity of microbial networks of karst tiankeng soils.


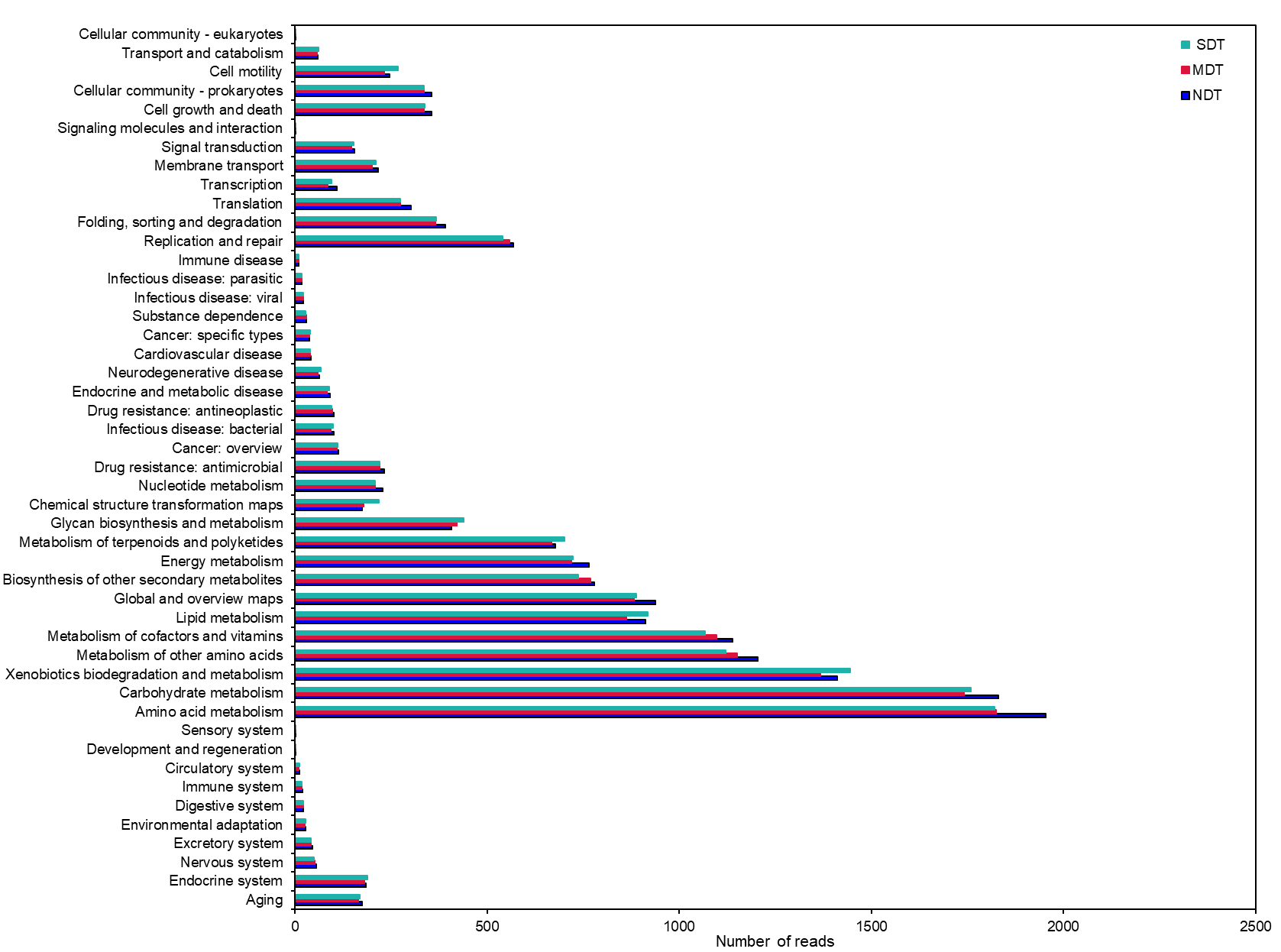


Fig. S5. The predicted KEGG categories abundance and percentage of karst tiankeng soils.


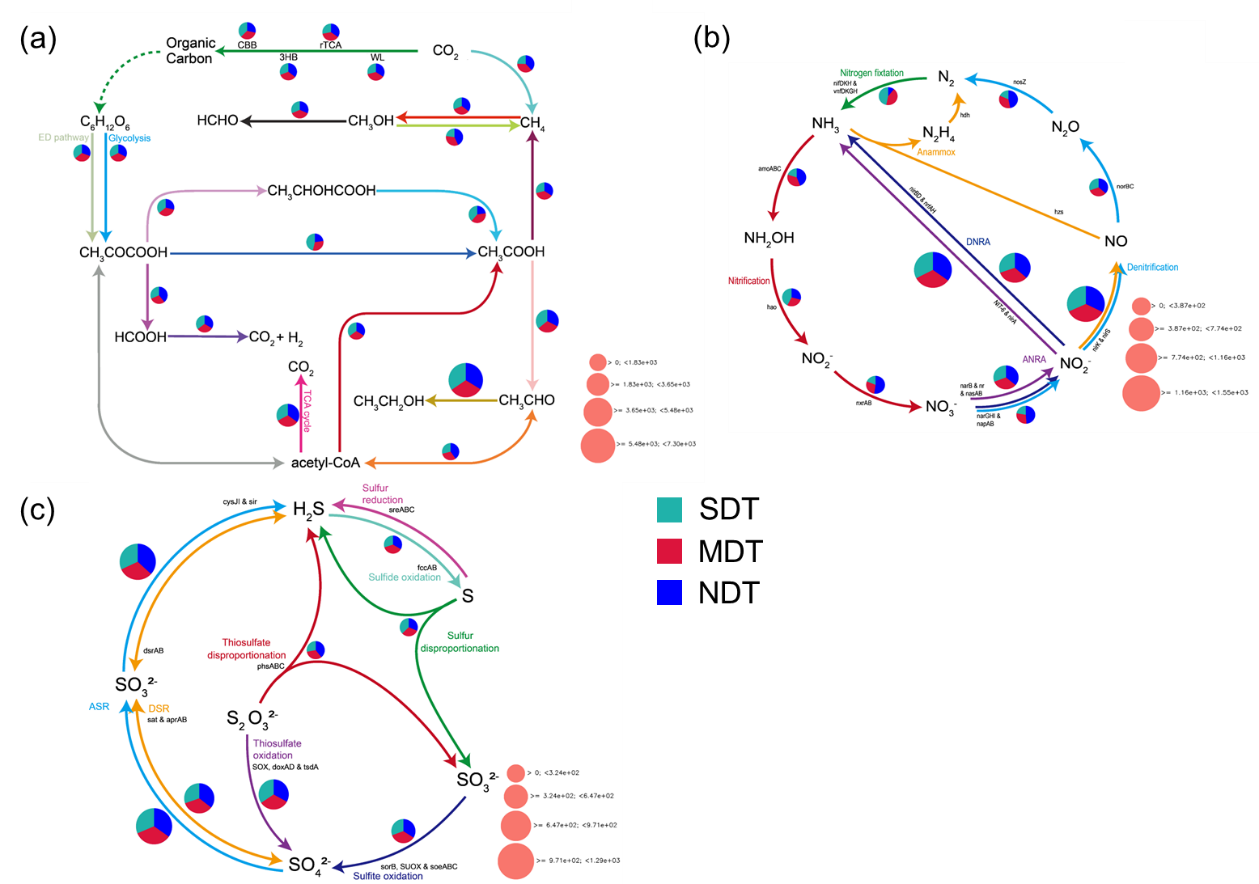


Fig S6. Relative abundances of the pathways involved in the carbon (a), nitrogen (b), and sulfur (c) cycle. The pie chart indicates the relative abundance of each pathway in each soil sample. The size of pie charts represent the total relative abundance of each pathway. Carbon cycle: CBB, Calvin-Benson-Bassham cycle; rTCA, reductive citric acid cycle; WL, Wood-Ljungdahl pathway; 3HB, 3-hydroxypropionate bicycle; DHC, dicarboxylate-hydroxybutyrate cycle. Nitrogen cycle: ANAR, assimilatory nitrate reduction to ammonium; DNRA, dissimilatory nitrate reduction to ammonium; Anammox, anaerobic ammonium oxidation. Sulfur cycle: ASR, assimilatory sulfate reduction; DSR, dissimilatory sulfate reduction.


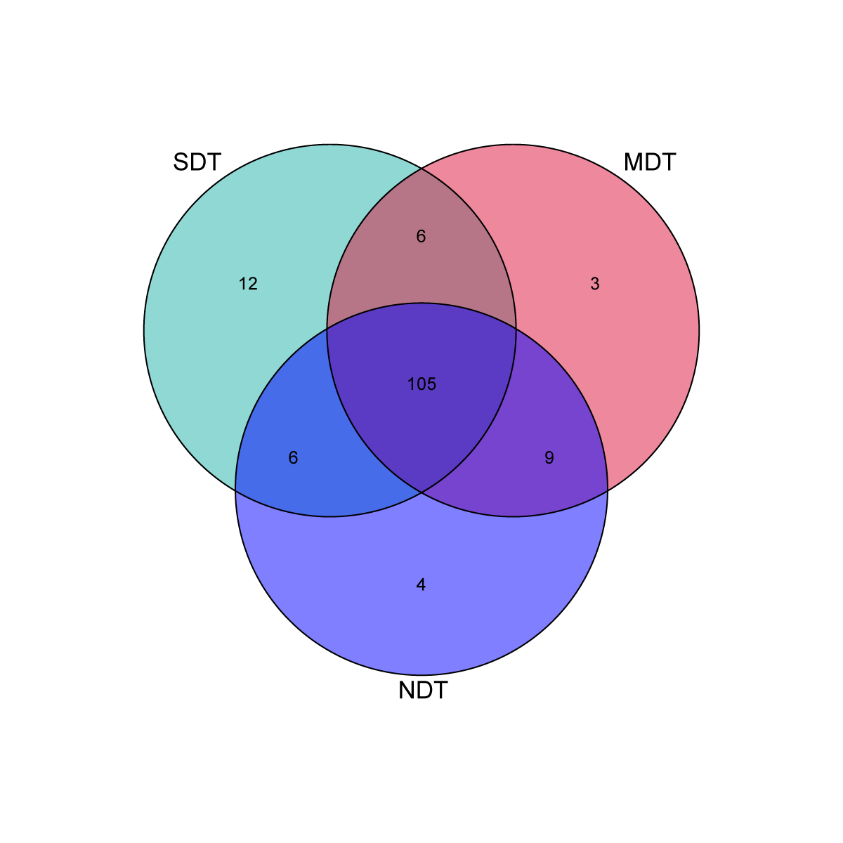


Fig. S7. Venn diagram of ARGs diversity of karst tiankeng soils.


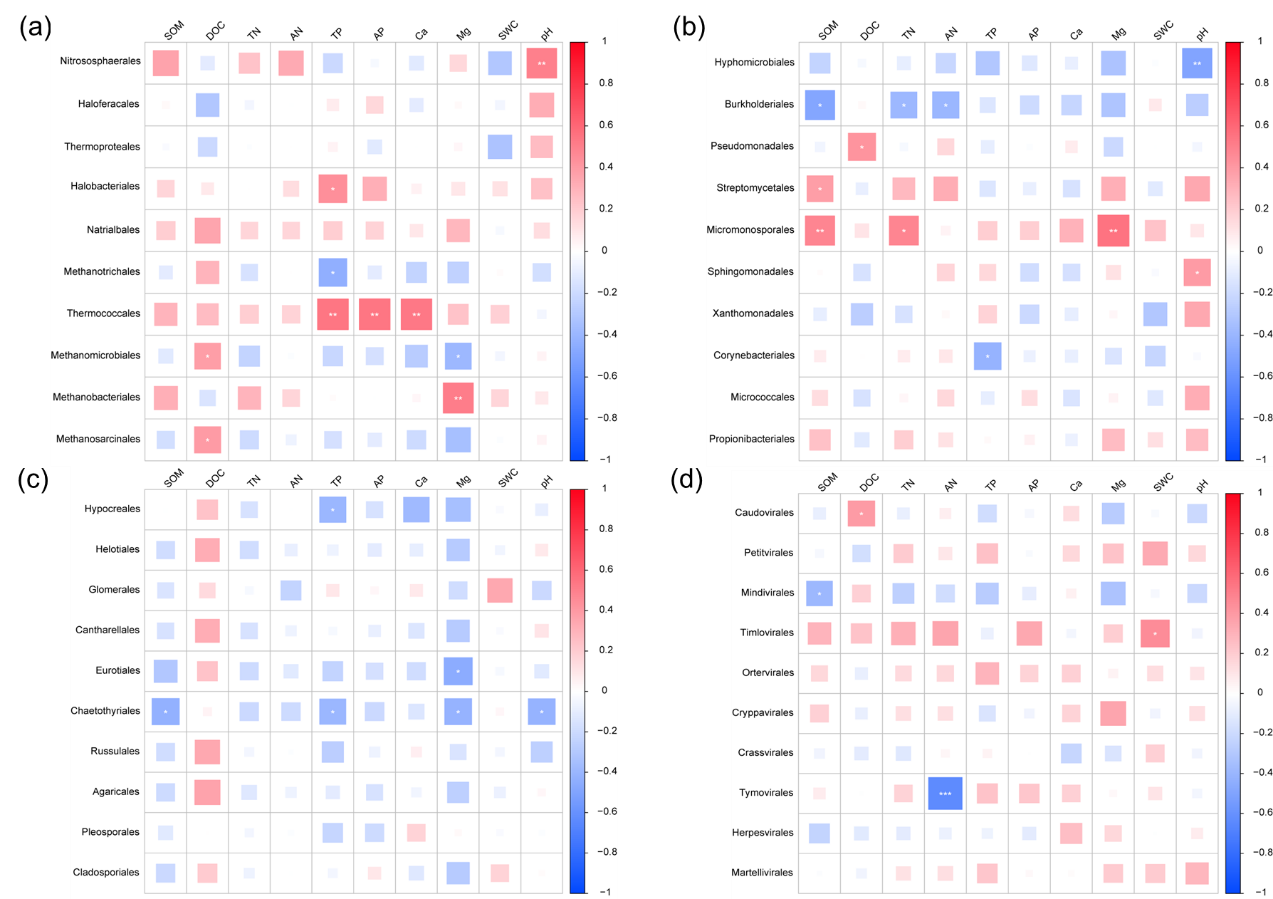


Fig. S8. Correlation between microorganisms and soil physicochemical properties of karst tiankeng (a: Archaea, b: Bacteria, c: Fungi, d: Viruses).

Table. S1. The key topological features of karst tiankeng soil microbial networks

|  | Nodes | Edges | P/N ratio | Average degree | Modularity | Average path length |
| --- | --- | --- | --- | --- | --- | --- |
| SDT | 178 | 119 | 77.31 | 1.34 | 0.98 | 1.44 |
| MDT | 18 | 9 | 77.78 | 1.00 | 0.89 | 1.00 |
| NDT | 98 | 58 | 84.48 | 1.18 | 0.96 | 1.37 |

P/N ratio means the ratio of positive edge and negative edge.
